# Genomic Characterization and Distribution Pattern of a Novel Marine OM43 Phage

**DOI:** 10.3389/fmicb.2021.651326

**Published:** 2021-03-24

**Authors:** Mingyu Yang, Qian Xia, Sen Du, Zefeng Zhang, Fang Qin, Yanlin Zhao

**Affiliations:** Fujian Provincial Key Laboratory of Agroecological Processing and Safety Monitoring, College of Life Sciences, Fujian Agriculture and Forestry University, Fuzhou, China

**Keywords:** OM43, OM43 phage, genomics, metagenomic viral genomes, distribution patterns

## Abstract

Bacteriophages have a significant impact on the structure and function of marine microbial communities. Phages of some major bacterial lineages have recently been shown to dominate the marine viral communities. However, phages that infect many important bacterial clades still remained unexplored. Members of the marine OM43 clade are methylotrophs that play important roles in C1 metabolism. OM43 phages (phages that infect the OM43 bacteria) represent an understudied viral group with only one known isolate. In this study, we describe the genomic characterization and biogeography of an OM43 phage that infects the strain HTCC2181, designated MEP301. MEP301 has a genome size of 34,774 bp. We found that MEP301 is genetically distinct from other known phage isolates and only displays significant sequence similarity with some metagenomic viral genomes (MVGs). A total of 12 MEP301-type MVGs were identified from metagenomic datasets. Comparative genomic and phylogenetic analyses revealed that MEP301-type phages can be separated into two subgroups (subgroup I and subgroup II). We also performed a metagenomic recruitment analysis to determine the relative abundance of reads mapped to these MEP301-type phages, which suggested that subgroup I MEP301-type phages are present predominantly in the cold upper waters with lower salinity. Notably, subgroup II phages have an inverse different distribution pattern, implying that they may infect hosts from a distinct OM43 subcluster. Our study has expanded the knowledge about the genomic diversity of marine OM43 phages and identified a new phage group that is widespread in the ocean.

## Introduction

Viruses play an important role in the marine microbial loop and biogeochemical cycles (Fuhrman, 1999; Suttle, 2005, 2007). They are the most abundant and genetically diverse biological entities in the ocean, with average abundance of an order of magnitude more than that of bacteria (Suttle, 2007; Güemes et al., 2016). Most marine viruses are bacteriophages that infect bacteria (Fuhrman, 1999; Wommack and Colwell, 2000). Bacteriophages can modulate the structure and function of the bacterioplankton communities since they serve as an important driver of bacterial mortality (Breitbart, 2012; Thingstad et al., 2014). Bacteriophages can also drive the evolution of bacteria *via* selective pressure (Marston et al., 2012; Martiny et al., 2014). Owing to the tremendous advance in cultured-independent technologies, such as metagenomics (Hurwitz and Sullivan, 2013; Mizuno et al., 2013; Brum et al., 2015; Paez-Espino et al., 2016; Gregory et al., 2019; Beaulaurier et al., 2020; Luo et al., 2020) and single-cell genomics (Roux et al., 2014; Labonté et al., 2015; Martinez-Hernandez et al., 2017), a comprehensive understanding of the viral community structure and diversity has emerged and many viral genome fragments have been obtained from these analyses. Compared to the thriving metagenomic studies, fewer studies describe the isolation and study of new marine phages. Therefore, most metagenomic viral sequences lack cultured counterparts that hamper the understanding of the ecological roles and biological traits of marine viruses. In recent years, bacteriophages that infect ecologically important and abundant marine bacterioplankton have received increasing attention. For example, pelagiphages, RCA phages and SAR116 phages have been shown to be diverse and dominate the viral communities (Kang et al., 2013; Zhao et al., 2013, 2019; Zhang et al., 2019, 2021; Buchholz et al., 2021). Other than the above-mentioned phages, phages infecting many important marine bacteria still remain poorly investigated.

The OM43 clade affiliated to the Type I methylotrophs of the family *Methlophilaceae* is known as an important component of marine coastal bacterial communities (Rappé et al., 2000; Suzuki et al., 2004), where it is commonly associated with phytoplankton blooms (Morris et al., 2006). OM43 clade has diverse metabolic profiles and plays an important role in the metabolism of the C1 compounds in the marine environments (Giovannoni et al., 2008; Halsey et al., 2012; Jimenez-Infante et al., 2016). Bacteria of this clade are difficult to culture in laboratories, owing to their sensitivity to slight biochemical variations of the seawater (Giovannoni et al., 2008), for this reason, there are limited OM43 strains have been isolated and genomically characterized so far. (Giovannoni et al., 2008; Huggett et al., 2012; Jimenez-Infante et al., 2016). To date, only one OM43 phage has been reported, limiting our understanding on their diversity, evolution, ecology, and impacts. Venkman, the first OM43 phage isolated on the OM43 strain H5P1, was isolated from the Western English Channel (Buchholz et al., 2021). Venkman exhibits significant genomic similarity with freshwater *Methylophilales* LD28 phage P19250A (Buchholz et al., 2021). HTCC2181 was the first isolate from the OM43 clade, which served as a good example of bacterioplanktons with reduced genome size to adapt to the nutrition limited environments (Giovannoni et al., 2008; Halsey et al., 2012). Although it is one of the most important model strain of the OM43 clade, HTCC2181 has no reported phages to date.

In this study, we describe the isolation, genomic characterization, and global distribution pattern of MEP301, a phage that infect OM43 strain HTCC2181. Genomic analyses reveal that MEP301 belongs to a novel phage group with all known relatives from phages assembled from metagenomic studies. We also identified some MEP301-type genomes from marine metagenomic datasets and conducted a metagenomic recruitment analysis to illustrate the global distribution patterns of MEP301-type phages.

## Materials And Methods

### Cultivation of OM43 strain HTCC2181

The axenic OM43 strain HTCC2181 was first isolated from the Oregon coast (Giovannoni et al., 2008) and was kindly provided by Prof. Stephen Giovannoni, Oregon State University. HTCC2181 was grown in seawater-based medium supplemented with 100 μM methanol, 1 mM NH_4_Cl, 100 μM KH_2_PO_4_, 1 μM FeCl_3_, and excess vitamins (Giovannoni et al., 2008). HTCC2181 cultures were incubated at 23°C in the dark without shaking.

### Source Waters and Phage Isolation

The water sample used for phage isolation was collected at Yantai coast, Bohai sea in China (N37°28, E121°28'). The seawater sample was filtered through 0.1-μm-pore-size filters to remove non-viral components. This cell-free sample was then stored at 4°C for further experiments. The details of phage isolation have been described in previous studies (Zhao et al., 2013; Zhang et al., 2019). Briefly, the filtered seawater samples were inoculated with exponential phase HTCC2181 culture and a Guava EasyCyte flow Cytometer was used to monitor the lysis of cell culture (Merck Millipore, Billerica, MA, United States). The presence of phage particles was confirmed by epifluorescence microscopy after cell lysis was detected (Suttle and Fuhrman, 2010). Purified phage clones were obtained by the dilution-to-extinction method (Zhao et al., 2013; Zhang et al., 2019), and the purity of the phage was verified by genome sequencing.

### Transmission Electron Microscopy

The lysate of MEP301 was filtered through a 0.1-μm filter and then centrifuged by ultracentrifugation (Beckman Coulter, United States) at 50,000 *g* for 2 h. A drop of the concentrated phage sample was then placed on a copper grid and subsequently dried in the air. The grid was stained for 2 min in 2% uranyl acetate and them observed by a Hitachi transmission electron microscope at a voltage of 80 kV.

### Phage DNA Preparation, Genome Sequencing, and Annotation

The phage lysate was filtered through 0.1-μm filters (Pall Life Sciences) to remove cell debris. Filtered phage lysate was concentrated to approximately 300 μl by using Amicon Ultra Centrifugal Filters (30 kDa, Merck Millipore) and Nanosep 10 K centrifugal tubes Ultra-centrifugal tubes (30 kDa, Pall Life Sciences). Phage DNA was extracted by the method of phenol-chloroform extraction (Sambrook et al., 1989). For Illumina sequencing, the DNA sample was sheared by Covaris M220 (Covaris, United States), and the DNA library was prepared using the NEBNext® Ultra™ DNA Library Prep Kit for Illumina (NEB, United States). Approximately 10 ng of the DNA sequencing library was used to generate a cluster in cBot using a TruSeq PE Cluster Kit (Illumina, United States) and whole genome sequencing of MEP301 was conducted using Illumina HiSeq™ 4,000 plateform (paired-end technology 2 × 150 bp) at Shanghai Hanyu Bio-Tech (Shanghai, China). The CLC Genomic Workbench 11.0.1 software (Qiagen, Hilden, Germany) was used to perform the quality control, trimming and *de novo* genome assembly with default parameters. To complete the whole genome, PCR amplification and Sanger sequencing were used to close gaps. The genome sequence of MEP301 has been deposited in the GenBank database under the accession number MW452941.

Putative ORFs in the MEP301 genome were identified using GeneMark (Besemer and Borodovsky, 1999). The translated ORFs were used as BLASTP queries to search against the NCBI database (e-value ≤ 1e-3, alignment coverage ≥50%, ≥25% amino acid identity). Putative functions were assigned to ORFs based on their homology to proteins of known function. The conserved domains of proteins were predicted by searching the PFAM database with Hmmer web server (Potter et al., 2018). Distant protein homologs were predicted by HHpred server (Söding et al., 2005). tRNA genes were searched by the tRNAscan-SE software (Lowe and Eddy, 1997).

### Retrieval of MEP301-Type Metagenomic Viral Genomes (MVGs)

To recover MEP301-type MVGs, the amino acid sequences of MEP301 ORFs were searched against 515,588 MVG sequences from Global Ocean Viromes (GOV and GOV2.0) (Roux et al., 2016; Gregory et al., 2019), the MedDCM fosmid library (Mizuno et al., 2013) and Station ALOHA (Beaulaurier et al., 2020) using BLASTP (e-value ≤ 10^−3^, ≥25% amino acid identity). Orthologous groups were determined using OrthoMCLv2.0 (Fischer et al., 2011). The contigs that share ≥40% genes with MEP301 and have a size ≥25 kb were designated as MEP301-type MVGs.

### Phylogenetic Analysis and Whole-Genome Phylogeny Analysis

A maximum likelihood phylogenetic tree of TerL protein sequences was constructed. Amino acid sequence alignment and editing were performed using Mafft and (Katoh et al., 2009) trimAl (Capella-Gutierrez et al., 2009), respectively. The phylogenetic tree was constructed using IQ-TREE v1.6.12 with 1,000 bootstrap replicates. Whole-genome based phylogeny based on amino acid sequences was built using VICTOR (Meier-Kolthoff and Göker, 2017).^3^ Genome-based classification at the genus and family level was performed using the OPTSIL program (Göker et al., 2009).

### Metagenomic Read Mapping

Marine viromic datasets that were downloaded to evaluate the relative abundance of MEP301-type sequences include Pacific Ocean Virome (POV) (Hurwitz and Sullivan, 2013), Malaspina Expedition Virome (MEV) (Roux et al., 2016), GOV (Roux et al., 2016), and GOV2.0 (Gregory et al., 2019). The MEP301-type sequences were first clustered at 70% average nucleotide identity and only the longest MVGs within a cluster was retained for recruitment analysis. Viromic reads (≥50 bp) were recruited using BLASTn as described (Mizuno et al., 2016; Martinez-Hernandez et al., 2017; Kim et al., 2019; López-Pérez et al., 2019; Zaragoza-Solas et al., 2020), with ≥70% identity and ≥90% read coverage. The relative abundances of phage genomes were normalized by total recruited nucleotides (kb) per kb of genome per gigabase of metagenome (KPKG). MEP301-type genomes for which <40% of the genomes was covered by recruited reads in a given viromic dataset were given a RPKG value of 0 (Buchholz et al., 2021).

## Results and Discussion

### Isolation and Morphology of MEP301

**Figure 1:**
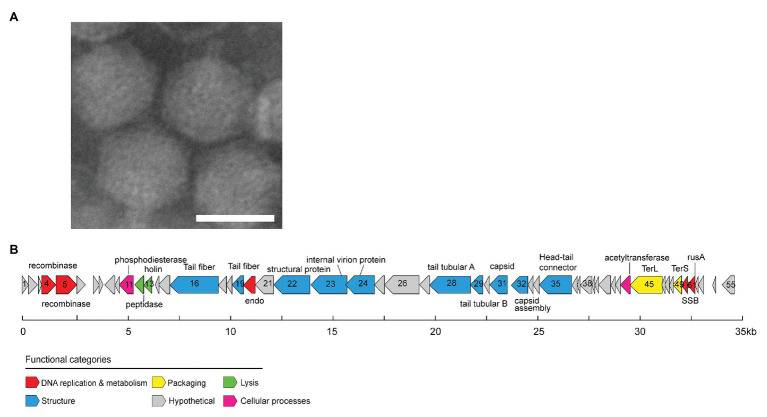
Transmission electron microscopy image of MEP301 **(A)**. Genome map of MEP301 **(B)**. Open reading frames (ORFs) are indicated by arrows with the arrow denoting the direction of transcription. ORFs are color-coded according to the putative biological function.

OM43 phage MEP301 was isolated from coastal surface water of Yantai, Bohai sea, China (N37°28', E121°28'). The TEM image shows that MEP301 is a phage with an icosahedral capsid of 59 ± 2 nm in diameter and a short tail (about 7 nm in length; Figure 1A). The morphological characteristics of MEP301 suggest that it belongs to a yet unassigned family of the *Caudovirales* order.

### General Genome Characteristics of MEP301

The genome of MEP301 was assembled into a single circular contig. This indicated that the sequence obtained represents a complete genome and suggested that its linear genome is either circularly permuted or has terminal repeats. MEP301 has a dsDNA genome of 34,774 bp in size. The G+C content of MEP301 genome is 40.4%, which was similar to that of its host HTCC2181 (37.9%) but significantly higher than that of the first OM43 phage Venkman (31.9%). A total of 55 open reading frames (ORFs) were identified in the MEP301 genome (Figure 1B). The BLASTP analysis revealed that the majority of the ORFs in MEP301 are most similar to genes found in metagenomic sequences. In addition, MEP301 does not display substantial genome similarity with any cultured phages. Overall, the above analyses suggest that MEP301 represents a new group of phages.

Approximately half of the ORFs in MEP301 can be assigned with predicted biological functions. These ORFs encode proteins associated with the DNA metabolism, virion structure, DNA packaging, and host lysis. No tRNA was identified in MEP301. Despite the universal metabolisms reprograming in host-phage interactions, it remains to be verified whether MEP301 rewires the host cellular metabolisms to utilize host specific tRNAs to synthetize viral proteins during infection.

### DNA Replication and Metabolism Related Genes

A total of five genes involved in DNA replication and metabolism have been identified from the MEP301 genome (Figure 1B). This includes genes encoding the N terminal resolvase domain protein (ORF4), YqaJ viral recombinase family protein (ORF5), single-stranded DNA (ssDNA) binding protein (SSB, ORF50), crossover junction endodeoxyribonuclease RusA superfamily protein (ORF51), and endonuclease (ORF20). Neither RNA polymerase nor DNA polymerase was identified in the MEP301 genome, suggesting that MEP301 is highly dependent on the host’s machinery for transcription and DNA replication.

ORF4 encodes a protein containing a N terminal resolvase domain (PF00239). In serine integrase, this domain is always associated with a catalytic recombinase PF07508 domain (Smith et al., 2010; Ambroset et al., 2016). However, no protein containing the PF07508 domain was detected in MEP301, indicating that MEP301 may not contain a functional serine recombinase. ORF5 encodes a YqaJ viral recombinase family protein (PF09588). This protein shares weak sequence identity with *Geobacillus* phage GBK2 and *Bacillus* phage SPP1 encoded exonuclease/recombinase (23 and 22% amino acid identity, respectively). This protein in phage SPP1 was reported to function as an alkaline exonuclease (Vellani and Myers, 2003). The SSB gene (ORF50) in the MEP301 genome is most similar to SSB genes from uncultured phage sequences, and also has similarity to those of some siphoviruses. For example, it shares 32 and 33% amino acid identity with those in *Acinetobacter* phage SH-Ab and DMU1, respectively. SSB proteins are ubiquitous in phage genomes. SSB proteins prevent ssDNA forming dsDNA by binding to the ssDNA and are essential for numerous DNA metabolic processes (Shereda et al., 2008).

Two genes involved in DNA cleavage have been identified including the crossover junction endodeoxyribonuclease RusA gene (ORF51, PF05866) and the predicted endonuclease gene (ORF20). Both gene products are predicted to cut the phosphodiester bonds between nucleotides in the interior DNA helix, resulting in the production of nucleotide monomers. MEP301 RusA shares 37.4% amino acid identity with that in *Shigella* phage 75/02 Stx. The RusA protein is an endonuclease that functions as a holliday junction resolvase and has been found to mediate the genetic recombination and repair in the prophage of *Escherichia coli* K-12 (Bolt et al., 1999). The predicted endonuclease shares 39.0% amino acid identity with that in *Pseudomonas* phage JG004. Thus, these two genes could presumably be involved in degrading host genomes to obtain sufficient nucleotide monomers for phage DNA synthesis, which is fundamental for phage reproduction.

### Structural Genes and DNA Packaging Genes

A set of structural genes, including genes encoding phage head-tail connector, capsid assembly protein, major capsid protein, internal virion proteins, and some tail related proteins, were predicted from the MEP301 genome (Figure 1B). Proteins encoded by these genes play important roles in phage morphogenesis. Most of these structural genes show higher sequence similarities with uncultured environmental viral genomes than to known phages, highlighting the novelty of MEP301. Among all cultured phages, some structural genes in MEP301 are most related to marine pelagiphages or SAR116 phage HMO-2011 with limited sequence identity. For example, the tail tubular protein B (ORF28) shares 27.6% amino acid identity with that in pelagiphage HTVC010P. The head-tail connector (ORF35) shares 27.0% amino acid identity with that in pelagiphage HTVC010P. The putative major capsid protein (ORF31) shares 30.7% amino acid identity with that in pelagiphage HTVC010P. The tail fiber protein (ORF19) shares 35.3% amino acid identity with SAR116 phage HMO-2011. The internal virion protein (ORF23) shares 30.3% amino acid identity with that in pelagiphage HTVC011P.

Both the large and small subunits of terminase (TerL and TerS) genes were identified in MEP301. These two subunits are indispensable for packaging the genome of most tailed bacteriophages (Sun et al., 2012). The terminases recognize the DNA for packaging and have a nuclease activity that is responsible for creating the ends of the virion chromosome (Casjens et al., 2005). The most closely related relatives of MEP301 TerL gene in cultured phage genomes are from *Cronobacter* phage vB_CsaP_Ss1, *Dunaliella viridis* virus SI2, *Burkholderia* phage vB_BmuP_KL4, and BcepC6B, with approximately 40% amino acid identity, suggesting that MEP301 may use a similar DNA packaging strategy. The TerS gene (ORF49; PF03592) has also been identified but does not share homology with any known TerS gene.

### Lysis Gene

The peptidase M15A (Peptidase_M15_3; PF08291), encoded by ORF12, shares 55% amino acid identity with that in an unfinished *Methylophilales* phage HIM624-A (accession number: AFB70783.1). HIM624-A, which infects *Methylophilacea* HIMB624, is also an OM43 phage, but its genome is not completed yet.

### Genes Likely Involved in Some Cellular Processes

The MEP301 ORF11 is predicted to belong to the metallophosphoesterase superfamily (PF00149) and shares homology with the phosphoesterase gene in *Vibrio* phage Vp670 (47.3% amino acid identity). Calcineurin-like phosphoesterases are common in bacteria and archaea genomes and have also been identified in many phage genomes. Calcineurin-like phosphoesterases have hydrolase activity against diverse phosphorylated substrates. The function of MEP301-encoded phosphodiesterases is still unclear. The MEP301 ORF44 encodes an acetyltransferase. Acetyltransferases are involved in the acetylation of a wide variety of substrates and play important roles in a large number of bacterial biological functions. Glycosyltransferases have been identified in many phage genomes and presumed to confer protection to phage from host restriction endonucleases and play important roles in temperate phage-mediated host immunity and host serotype conversion (Markine-Goriaynoff et al., 2004). The specific role of MEP301 encoded acetyltransferase, however, remains unclear.

### MEP301-Type Metagenomic Viral Genome

**Figure 2:**
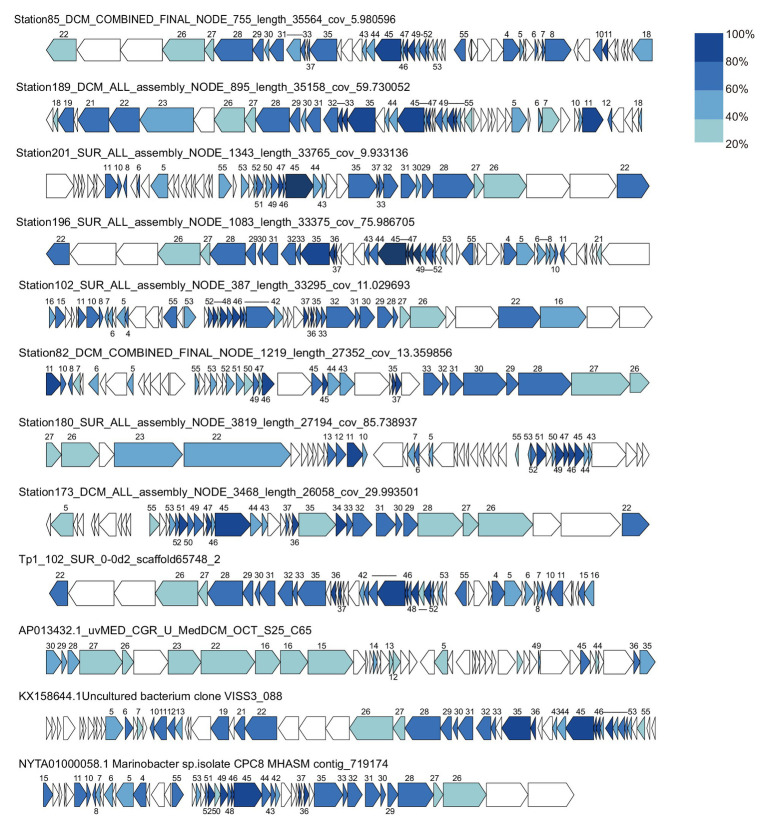
Genome organization of 12 metagenomic viral genomes (MVGs) related to MEP301. Open reading frames (ORFs) are colored according to the degree of amino acid sequence identity to the genes in MEP301. The number of MEP301 homologous ORFs are indicated above the frames.

**Figure 3:**
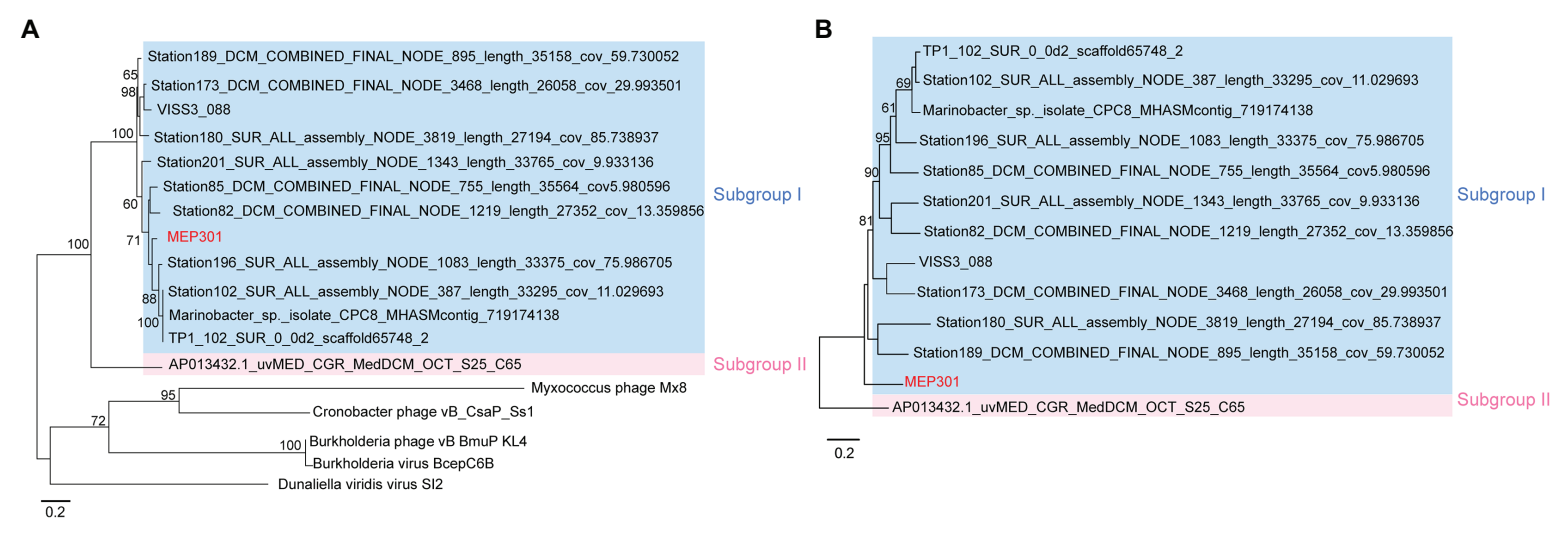
**(A)** Phylogenetic analysis of TerL protein sequence identified from MEP301 and 12 MEP301-type metagenomic viral sequences (MVGs). **(B)** Phylogenomic tree of the MEP301-type genomes at the amino acid levels constructed by VICTOR web service.

A search was performed to retrieve MEP301-type MVGs from environmental metagenomes. A total of 12 MEP301-type MVGs that share ≥40% genes with MEP301 were retrieved from various marine viromic datasets. These MEP301-type MVGs range in size from 26 to 35 kb, encoding 42 to 57 ORFs (Table 1). The G+C content of these MVGs range from 33.5 to 37.8%, similar to that of MEP301. Genomic comparison indicated that these 12 MVGs phages display obvious relationships with the MEP301 with sequence similarities and overall conservation of genome architectures (Figure 2). We classified these MVGs into the MEP301-type phage group. Compared to genes in other MVGs, corresponding genes in an uncultured Mediterranean phage fosmid sequence uvMED-CGR-U-MedDCM-OCT-S25-C65 have lower sequence similarity to MEP301 (23–65%, average 39.3%). To investigate the evolutionary relationships among these MEP301-type phage genomes, TerL protein were used for phylogenetic analysis. Based on the TerL phylogeny, these 12 MVGs are clustered with MEP301 and form a separate branch, indicating that MEP301-type is a novel phage group. All MEP301-type genomes were further separated into two major subgroups with subgroup I containing 11 genomes and subgroup II containing uvMED-CGR-U-MedDCM-OCT-S25-C65 (Figure 3A). The whole genome-based VICTOR phylogeny also shows a similar topology (Figure 3B). The OPTSIL taxon prediction suggests that these MEP301-type phages belong to a subfamily-level group and can be separated into two genus-level subgroups. Collectively, these results confirm that these MVGs belong to the same phage group with MEP301, and MEP301-type phage group containing at least two closely-related subgroups.

**Table 1:** Genomic features of 12 nearly full-length MEP301-type MVGs.

MEP301-type MVGs	Length (bp)	G+C%	Source
Station85_DCM_COMBINED_FINAL_NODE_755_length_35564_cov_5.980596	35,564	36.6	Antarctic province
Station189_DCM_ALL_assembly_NODE_895_length_35158_cov_59.730052	35,158	34.9	Boreal polar province
Station201_SUR_ALL_assembly_NODE_1343_length_33765_cov_9.933136	33,765	36.7	Boreal polar province
Station196_SUR_ALL_assembly_NODE_1083_length_33375_cov_75.986705	33,375	37.6	Boreal polar province
Station102_SUR_ALL_assembly_NODE_387_length_33295_cov_11.029693	33,295	37.8	Pacific equatorial divergence province
Station82_DCM_COMBINED_FINAL_NODE_1219_length_27352_cov_13.359856	27,352	33.5	Southwest Atlantic shelves province
Station180_SUR_ALL_assembly_NODE_3819_length_27194_cov_85.738937	27,194	38.6	Boreal polar province
Station173_DCM_ALL_assembly_NODE_3468_length_26058_cov_29.993501	26,058	35.0	Boreal polar province
Tp1_102_SUR_0-0d2_scaffold65748_2	33,618	37.6	Pacific equatorial divergence province
uvMED-CGR-U-MedDCM-OCT-S25-C65	32,359	37.1	Mediterranean Sea deep chlorophyll maximum
KX158644.1 Uncultured bacterium clone VISS3_088	34,150	36.4	East Greenland current (station 3) of the boreal polar region
NYTA01000058 MHASMcontig_719174138	33,650	37.8	Indian monsoon gyres province

### Global Distribution of MEP301-Type Phages

**Figure 4:**
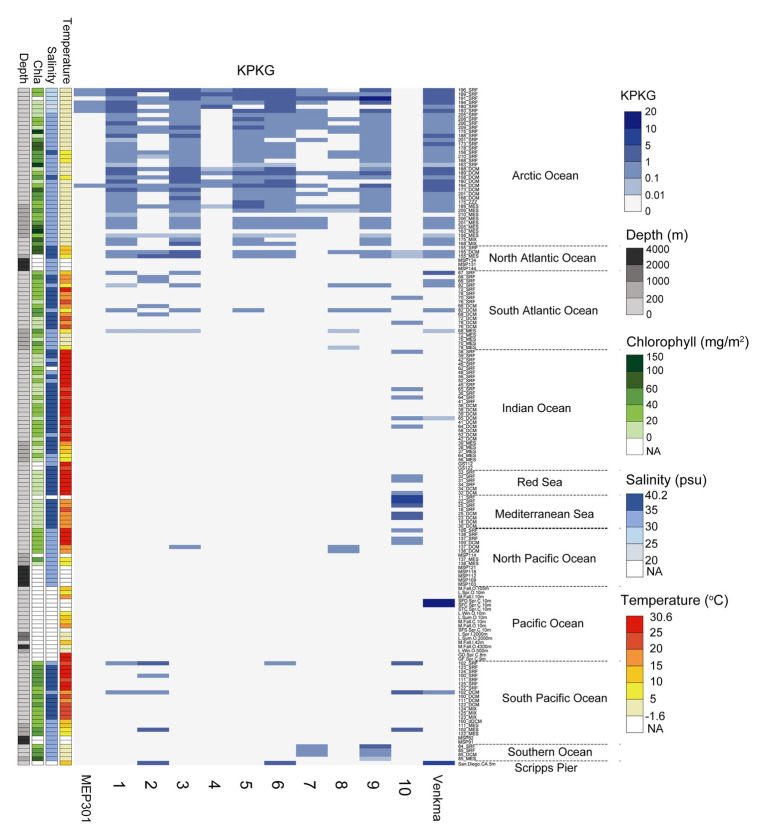
Heatmap displaying the relative abundance of each MEP301-type phage in different marine viromic datasets. Normalized relative abundance is depicted as total mapped nucleotides (kb) per kb of genome per gigabase of metagenome (KPKG). The number (1–10) on the x-axis represent the MEP301-type phage: 1. KX158644.1Uncultured_bacterium_clone_VISS3_088, 2. Station102_SUR_ALL_assembly_NODE_387_length_33295_cov_11.029693, 3. Station173_DCM_ALL_assembly_NODE_3468_length_26058_cov_29.993501, 4. Station180_SUR_ALL_assembly_NODE_3819_length_27194_cov_85.738937, 5. Station189_DCM_ALL_assembly_NODE_895_length_35158_cov_59.730052, 6. Station196_SUR_ALL_assembly_NODE_1083_length_33375_cov_75.986705, 7. Station201_SUR_ALL_assembly_NODE_1343_length_33765_cov_9.933136, 8. Station82_DCM_COMBINED_FINAL_NODE_1219_length_27352_cov_13.359856, 9. Station85_DCM_COMBINED_FINAL_NODE_755_length_35564_cov_5.980596, 10. uvMED−CGR−U−MedDCM−OCT−S25−C65, respectively.

We next queried for the presence of MEP301-type phages in publicly available marine metagenomes by mapping viromic reads to MEP301-type genomes. Viromic read mapping (≥70% nucleotide identity) revealed that these MEP301-type phages can be detected in many oceanic stations (Figure 4), but their relative abundance was significantly low compared to the relative abundance of previously reported pelagiphages (Zhang et al., 2019). These phages were found to be distributed mainly in surface ocean waters (>200 m). In surface waters, all subgroup I genomes were found predominantly in the cold waters of Arctic Ocean and North Atlantic Ocean, where both temperature and salinity were lower (Figure 4). These oceanic regions also displayed higher chlorophyll values. They were also detected in some stations of South Atlantic, Pacific Ocean, and Southern Ocean. We also noticed that the first sequenced OM43 phage Venkman had a similar distribution pattern with subgroup I MEP301-type phages. Interestingly, subgroup II genome uvMED_CGR_U_MedDCM_OCT_S25_C65 displayed a distinct distribution pattern. It was not detected in Arctic Ocean stations but was detected in other analyzed oceanic regions, where temperature and salinity were higher. Phylogenetically, uvMED_CGR_U_MedDCM_OCT_S25_C65 is more distantly related to other MEP301-type phages. Their different distribution pattern may be due to differences in host species. The OM43 clade can be separated into two divergent clusters with different distribution pattern (Jimenez-Infante et al., 2016). HTCC2181-cluster has high abundance in high chlorophyll content but lower temperature waters, while the H-RS cluster is more abundant in warmer and high salinity waters (Jimenez-Infante et al., 2016). Generally, the distribution of a phage is broadly correlated to the distribution of its hosts. Based on these observations, it is likely that the subgroup II phage infect hosts from the H-RS cluster.

## Conclusion

In this study, we sequenced and analyzed the genome of a new OM43 phage MEP301 and obtained related phage sequences from viromic datasets, providing new insights into the diversity and evolution of marine OM43 phages. We also provided new insights into the distribution patterns of this important phage and highlight their ecological and evolutionary relevance. We showed that MEP301-type phages are widely distributed in marine environments although they were not in high abundance. Our results raise questions for future studies on the influence of MEP301-type phages on OM43 clade physiology and diversification. Phage MEP301 can serve as a model system for the study of OM43 phage ecological roles and phage-host interactions.

## Data Availability Statement

The datasets presented in this study can be found in online repositories. The names of the repository/repositories and accession number(s) can be found in the article/Supplementary Material.

## Author Contributions

MY and QX performed the genomic analyses and prepared the manuscript. SD performed the metagenomic analysis. ZZ contributed in bioinformatics analysis. FQ isolated the phage. YZ designed the study and edited the manuscript. All authors contributed to the article and approved the submitted version.

### Conflict of Interest

The authors declare that the research was conducted in the absence of any commercial or financial relationships that could be construed as a potential conflict of interest.

## Footnotes

^1^https://ggdc.dsmz.de/victor.php
